# Application of *COI*-LAMP for Detection of *Dirofilaria immitis* with High Sensitivity and Specificity in Epidemiological Studies

**DOI:** 10.1007/s11686-026-01253-w

**Published:** 2026-03-09

**Authors:** Melih Gazi Genc, Ufuk Erol, Omer Faruk Sahın, Kursat Altay

**Affiliations:** 1 Department of Veterinary Parasitology, Institute of Health Sciences, Sivas Cumhuriyet University, Sivas, Turkey; 2 Department of Parasitology, Faculty of Veterinary Medicine, Sivas Cumhuriyet University, 58140 Sivas, Turkey

**Keywords:** *Dirofilaria immitis*, Dog, Cat, PCR, DNA sequence analysis, LAMP, Türkiye

## Abstract

**Purpose:**

*Dirofilaria immitis*, a mosquito-borne zoonotic nematode, has worldwide distribution and causes infections in domestic and wild animals. Microscopic, serological, and molecular diagnostic methods are used to investigate this parasite in the hosts. Molecular diagnostic methods are outstanding for their sensitivity and specificity. The LAMP method, which has been used in detecting many parasites with its high specificity and sensitivity in recent years, is also advantageous with its simplicity of application. This study aimed to use the *COI*-LAMP method in the diagnosis of *D. immitis* in different host species.

**Methods:**

LAMP primers specific for the *COI* gene of *D. immitis* were designed, and the method was optimized. Additionally, the sensitivity, specificity, and limit of detection of the LAMP method were determined, and the results were compared with those of the PCR method. Moreover, to demonstrate the effectiveness of the LAMP method in epidemiologic studies, 600 blood samples were collected from dogs (n:300) and cats (n:300) in different parts of Türkiye. gDNA obtained from these samples were researched with LAMP and PCR assays, and the results were compered. Level of agreement between assays was calculated with Cohen’s kappa test.

**Results:**

The limit of detection of the LAMP method was determined to be 0.0048 ng/μL, while that of the PCR method was 0.48 ng/μL, indicating that the LAMP method was approximately 100 times more sensitive than PCR. The blood samples were examined in terms of *D. immitis*, and ten samples (1.66%) were found to be positive. In contrast, six samples (1%) were positive by PCR. *D. immitis* was detected in nine (3%) dogs and one (0.33%) cat by LAMP method, and this parasite was detected in six (2%) dogs by PCR. *Dirofilaria immitis* was not detected by PCR in cat samples. The kappa value was calculated as κ = 0.76; this result revealed that the “substantial” agreement between assays.

**Conclusion:**

Our results showed that *COI*-LAMP has high sensitivity in the diagnosis of *D. immitis* in different hosts. It was also understood that its use in epidemiological studies would be useful. Since it is critical to know more accurate epidemiological data in the fight against the disease, it will be useful to use more sensitive diagnostic methods, like LAMP, in studies to be conducted in this field.

**Supplementary Information:**

The online version contains supplementary material available at 10.1007/s11686-026-01253-w.

## Introduction

Parasitic pathogens have a global distribution, and these threaten animal and human health [1–3]. Millions of animals and thousands of people die every year in the world due to parasitic diseases [4, 5]. *Dirofilaria immitis* (family: Onchocercidae, genus: *Dirofilaria*) is known to be one of the important mosquito-borne filarioid nematodes, which affect both animal (domestic and wild) and human health [1, 2]. This nematode is reported mostly in countries that are located in tropical and subtropical regions [2, 6, 7]. *Dirofilaria immitis* causes mild to severe infection, and clinical infections are mostly associated with the number of adult parasites placed in the heart and pulmonary arteries and the number of L1 circulated into the bloodstream. Clinical symptoms such as fatigue, exercise intolerance, respiratory system disorders (such as cough), lack of appetite, epistaxis, jaundice, and ascites might be correlated with *D. immitis* infection in dogs. In some cases, *D. immitis* can cause death in dogs. Similar symptoms may also be seen in cats infected with *D. immitis*. Clinical manifestations in these animals are milder; however, the nematode may lead to death in cats [1, 7, 8].

Several laboratory techniques (microscopic, serologic, and molecular) have been used to identify *D. immitis* in hosts [9]. Microscopic examination of blood samples is widely used for the detection of microfilaria in the bloodstream. The advantages of microscopic techniques are that they are easy to apply and inexpensive. However, several disadvantages of these methods are present, such as low sensitivity and specificity, especially low amounts of microfilaria in the bloodstream. In such cases, animals with low parasite loads may be overlooked [6, 9, 10]. Serological methods, such as ELISA, IFA, and LAT, have been utilized for the diagnosis of *D. immitis* in hosts. However, antigen-ELISA has been mostly used by researchers due to its high sensitivity and specificity compared to other serologic and microscopic techniques [9]. This technique aims to detect circulating antigens produced by adult females. Antigen-ELISA can also detect occult *D. immitis* infection in hosts, and it is another advantages the method. However, there are some disadvantages of serological methods, like false positive results due to cross-reaction with other filarioid nematodes that may be present in the hosts and false negative results due to a low amount of antigen (remaining below the detection limit of the method) circulating in the hosts' blood systems [8–10]. If infected animals are missed using either microscopic or serological methods, the pathogen can be transmitted to other animals or humans in the surrounding area via mosquitos [6, 9, 10].

Molecular methods, like PCR, nested-PCR, and real-time PCR, have been successfully used for the identification of *D. immitis* in hosts. Both the sensitivity and specificity of these techniques are higher than microscopic and serologic methods [9]. Molecular methods have been preferred in various parts of the world due to their advantages. It is known that the most important disadvantage of these methods is the need for costly equipment (such as thermal cycler or real-time PCR devices) to perform the techniques. For this reason, PCR-based methods can only be applied in regions with relatively advanced laboratories [11, 12]. Different molecular methods have been developed to overcome these disadvantages of PCR-based molecular methods. One of these methods is the Loop-Mediated Isothermal Amplification (LAMP). LAMP is a molecular-based diagnostic method that amplifies target DNA under isothermal conditions in a single step with high sensitivity and specificity [11]. The advantages of the LAMP assay are that it does not require the expensive devices needed for other molecular methods, the method gives results in about an hour, and the results can be evaluated even with the naked eye [11–13].

Türkiye has a favorable climate for living vector species, like mosquitoes and ticks, and many vector species have been reported in the country. In addition, several vector-borne pathogens have been detected in animals and humans in almost all parts of Türkiye [14–16]. One of the important vector-borne pathogens is *D. immitis*, which has been reported in different parts of Türkiye. However, the number of studies conducted on *D. immitis* using molecular methods in Türkiye is considered to be limited compared to other vector-borne pathogens [17–20]. This study has three main objectives. The first is to develop and optimize a LAMP assay for the diagnosis of *D. immitis* in cats and dogs. The second is to investigate the presence and prevalence of *D. immitis* in cats and dogs using molecular methods in Istanbul, Ankara, and Izmir, which are Türkiye's most populous provinces. The third is to perform molecular characterization and phylogenetic analysis of samples determined to be positive.

## Materials and methods

### Determination of the COI-LAMP Primers for Identification of Dirofilaria Immitis

The Loop-Mediated Isothermal Amplification assay was developed to identify *D. immitis* in hosts. The *cytochrome oxidase subunit I* (*COI*) gene was chosen for this purpose. Before designing the LAMP primers, all nucleotide sequences belonging to the *D. immitis COI* gene were downloaded from GenBank. These sequences were aligned using the MEGA-11 program [21], and the *D. immitis* genotypes circulating in the world were determined. Sequences randomly selected to represent all genotypes (supplementary table) were used for the design of LAMP primers. LAMP primers that can detect all of these genotypes were designated (Table 1). These primer sets were determined using an online program (NEB® LAMP primer design tool https://lamp.neb.com/#!/). In-silico analyses were performed to determine whether the selected primer sets amplify different pathogens, such as *Dirofilaria repens, Acanthocheilonema reconditum, Wuchereria bancrofti, Onchocerca volvulus, Loa loa, Brugia malayi, B. timori, B. pahangi, Mansonella ozzardi, M. perstans,* and *M. streptocerca*, utilizing the BLASTn algorithm.

**Table 1:** LAMP primer sets used for the identification of *D. immitis*

Primers	Nucleotide sequence (5′–3′)
F3	TTGGTGGTTTTGGTAATT(G/T)G
B3	AAGATAACTCAGGCTGACC
FIP	ACGCAAC(A/G)(A/G)AAGTAATCCAAAAAGAGTTGCCTTTGATATTGGGTG
BIP	TCAATCTTTTTTTATTGG(G/A)GGGGGCTC(T/C)ACACTCAA(A/G)GG(A/G)GGAT
Loop F	GCATTAACACGAGGAAAAGCCATT
Loop B	CCTGG(G/T)AGTAGTTGAACTTT

### Optimization of Loop-Mediated Isothermal Amplification Method

The best incubation temperature of the LAMP method was determined. For this purpose, *D. immitis* positive DNA (GenBank accession number: KJ183078) obtained from infected dogs was used. The optimization process was carried out using NEB WarmStart Colorimetric LAMP 2× Master Mix (DNA&RNA) (Cat. No.: M1800S, New England BioLabs), following the instructor’s recommendations. LAMP master-mix was prepared in a total volume 25 μL using 9 μL water (DNAse, RNAse free), 12.5 μL 2 × LAMP Buffer, 1.6 μM FIP (100 μM), 1.6 μM BIP (100 μM), 0.2 μM F3 (100 μM), 0.2 μM B3 (100 μM), 0.8 μM LoopF (100 μM), 0.8 μM LoopB (100 μM), 1.6 μM FIP (100 μM), 1.6 μM BIP (100 μM), 0.2 μM F3 (100 μM), 0.2 μM B3 (100 μM), 0.4 μM LoopF (100 μM), 0.4 μM LoopB (100 μM), and 1μL template DNA.

To determine the best amplification temperature, LAMP master mixes were amplified for 60 min at different temperatures, such as 58.0 °C, 59.4 °C, 60.4 °C, 61.4 °C, 62.6 °C, 63.5 °C, and 65.0 °C. After this procedure, the LAMP master mixes were checked for color shift by the naked eye and agarose gel electrophoresis. The results were recorded.

### The Determination of Limit of Detection both LAMP and Conventional-PCR Method

To ascertain the limit of detection of the LAMP method, a sample obtained from an adult *D. immitis* of DNA was used. The amount of DNA in the samples was measured with a nanodrop (Ds-11, Denovix). This DNA was diluted using sterile water, and samples containing different amounts of pathogen DNA (10^0^→10^–7^) were obtained. These samples were used to determine the limit of detection of both the LAMP and PCR assays.

The limit of detection of the LAMP assay was determined by adding samples containing different amounts of *D. immitis* DNA to the master mix prepared as described in the section “Optimization of Loop-Mediated Isothermal Amplification Method”. After LAMP assay results were checked by both naked eyes and agarose gel electrophoresis, and the obtained results were recorded.

In this study, the limit of detection of the PCR assay was also determined using primers amplified 203 bp of the *COI* gene region of *D. immitis* DICOIF1 (5′-AGTGTAGAGGGTCAGCCTGAGTTA-3′) and DICOIR1 (5′-ACAGGCACTGACAATACCAAT-3′) [22]. The PCR mixture was prepared as detailed by Erol et al. [23]. PCR assay was done in the following protocol; first denaturation at 95 °C for 5 min., 35 cycles denaturation at 95 °C for 30 s., amplification at 55 °C for 30 s., and extension at 72 °C for 30 s., and final extension at 72 °C for 5 min. The PCR products were loaded onto the 1.5% agarose gel and subjected to electrophoresis at 90 V for 60 min. The agarose gel was stained with ethidium bromide for 20 min. The gel was controlled for positive and negative results using a UV transilluminator, and the results were recorded.

Additionally, to demonstrate the repeatability of the developed LAMP assay, the method was run on samples known to be PCR-positive (n:10) and PCR-negative (n:10) in terms of *D. immitis*. *Dirofilaria immitis* positive samples were checked using PCR for possible DNA degradation before being used in the repeatability analyses. The PCR assay was performed using DICOIF1 and DICOIR1 primers [22]. PCR and LAMP master mixes were prepared as above-mentioned. PCR and LAMP results were checked and recorded (supplementary figures).

### Molecular Investigation of the Epidemiology of Dirofilaria Immitis by LAMP and PCR

#### Study Area and Sample Collection

Türkiye is located between the temperate and subtropical zones. The country connects Europe and Asia and is a natural bridge between the two continents. Türkiye is one of the most populous countries in Europe, with a population of about 86 million. Approximately 22% of the country's population lives in Istanbul, Ankara, and Izmir [24, 25].

**Fig. 1:**
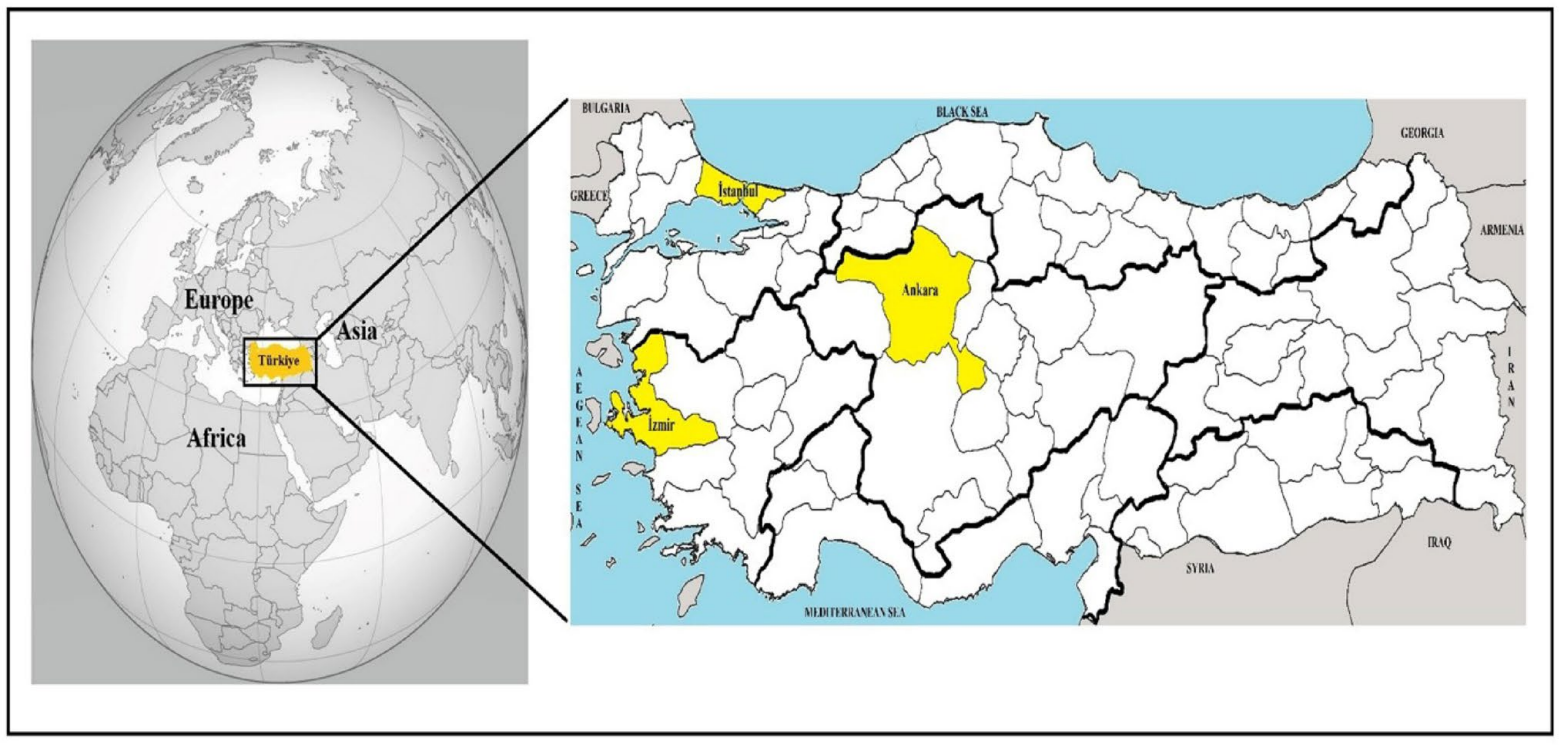
Location of Türkiye. Sampling areas were indicated with yellow on the Türkiye map

The study material consisted of 600 blood samples (300 cats and 300 dogs) from the provinces of Istanbul (cat: 100, dog: 100), Ankara (cat: 100, dog: 100), and Izmir (cat: 100, dog: 100) (Fig. 1). These samples were obtained from owned-animals brought to veterinary clinics and hospitals for different reasons (treatment, vaccination, and/or control, etc.) between 2023 and 2024. Blood samples were collected by veterinarians from the cephalic veins of the animals into EDTA-containing tubes. These tubes were stored at − 20 °C until used molecular methods.

#### Genomic DNA Extraction and Molecular Investigation

Genomic DNA was obtained from blood samples using a commercial extraction kit (Exgene™ Clinic SV (250 prep.) (Cat. No.: 108–152, Geneall®, South Korea), all procedures were done following the manufacturer’s instructions. Positive (GenBank accession number: KJ183078) and negative (sterile water) controls were used to prevent false positive and negative results during the gDNA extraction process. The obtained gDNAs were kept at − 20 °C until they were used.

Out of 600 DNA samples were researched in terms of *D. immitis* using both LAMP and PCR methods. LAMP assay was carried out using NEB WarmStart Colorimetric LAMP 2× Master Mix (DNA&RNA) (Cat. No.: M1800S, New England BioLabs), and amplification was done at 62 °C for 60 min. Controls [positive (GenBank accession number: KJ183078) and negative (sterile water)] were added to the LAMP assay to prevent negative and positive results. After the incubation period, the samples were categorized as positive or negative according to the observed color shift and agarose gel electrophoresis.

In the PCR assay for identification of the pathogen, the primers Di-F (5′-AGCTCGTAGTTGGATCTGCAT-3′) and Di-R (5′-CGTCAAGGCGTATTTACCG-3′) amplified 453 bp parts of *D. immitis 18S rRNA* gene were used [26]. The PCR assay was done with the cycling conditions described by Ataş et al. [19]. Positive (GenBank accession number: KJ183078) and negative (sterile water) controls were used in the PCR assay.

Since the LAMP method developed within the aim of this study is specific to the *COI* gene region, all positive samples were also researched using primers DICOIF1 and DICOIR1 that amplify the *COI* gene region of *D. immitis* [22], and the results were recorded.

To prevent cross-contamination during the laboratory process, DNA extraction, preparation of master mixes (both PCR and LAMP), addition of DNA to the mixes, loading of PCR and LAMP products onto 1.5% agarose gel, and gel visualization were performed in separate rooms.

### DNA Sequence and Phylogenetic Analyses of PCR-Positive Samples

All PCR-positive samples were sequenced with the primers amplifying to the *18S rRNA* gene of *D. immitis* [26]. Before DNA sequence analysis, the samples were purified with the MAGBIO ‘HighPrep™ PCR Clean-up System’ kit (Cat. No.: AC-60005). DNA sequencing was utilized with an ABI 3730XL Sanger sequencer (Applied Biosystems, Foster City, CA) using the ‘BigDye Terminator v3.1 Cycle Sequencing Kit’ (Applied Biosystems, Foster City, CA). Obtained DNA sequenced files open with FinchTV (version 1.4.0, Geospiza Inc., Seattle, Washington, USA) software, and chromatograms were checked for quality scores.

Forward and reverse sequence data were aligned with MEGA-11 software [21]. Nucleotides that had low-quality scores were trimmed, and consensus sequences were determined. The obtained consensus sequences were deposited to the GenBank, and accession numbers were obtained. The nucleotide similarities between our *D. immitis* isolates and *D. immitis* identified in different parts of the world were determined using the BLASTn algorithm present in the NCBI.

The phylogenetic tree was created using the Maximum Likelihood Algorithm in the MEGA-11 software [21] to specify the genetic relationship between our *D. immitis* isolates. Before constructing the phylogenetic tree, the Find Best-Fit Substitution Model function in MEGA-11 [21] was used to determine the best algorithm to be used in the phylogenetic tree. The Tamura-3 parameter model [27] was used to build the phylogenetic tree. The bootstrap analysis was carried out with 1000 replications.

### Statistical Analysis

Statistical analyses of the results obtained in the study were performed with Pearson chi-square test, and the data with *p* < 0.05 were considered statistically significant. Cohen's kappa test was used to evaluate the agreement between obtaining results utilizing conventional-PCR and LAMP assay in the samples [28]. Cohen’s kappa test was calculated following the formula;

$$ \kappa = \frac{\Pr (a) - \Pr (e)}{{1 - \Pr (e)}} $$

The results obtained after calculation were interpreted according to the values presented by Landis and Koch [29].

## Results

### Optimization of LAMP Method for the Diagnosis of D. immitis and the Determination of Limit of Detection

**Fig. 2:**
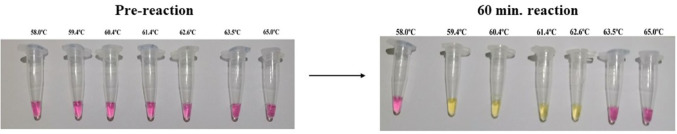
The determination of annealing temperatures of the LAMP method with the naked eye. Color change was seen after 60 min of reaction

**Fig. 3:**
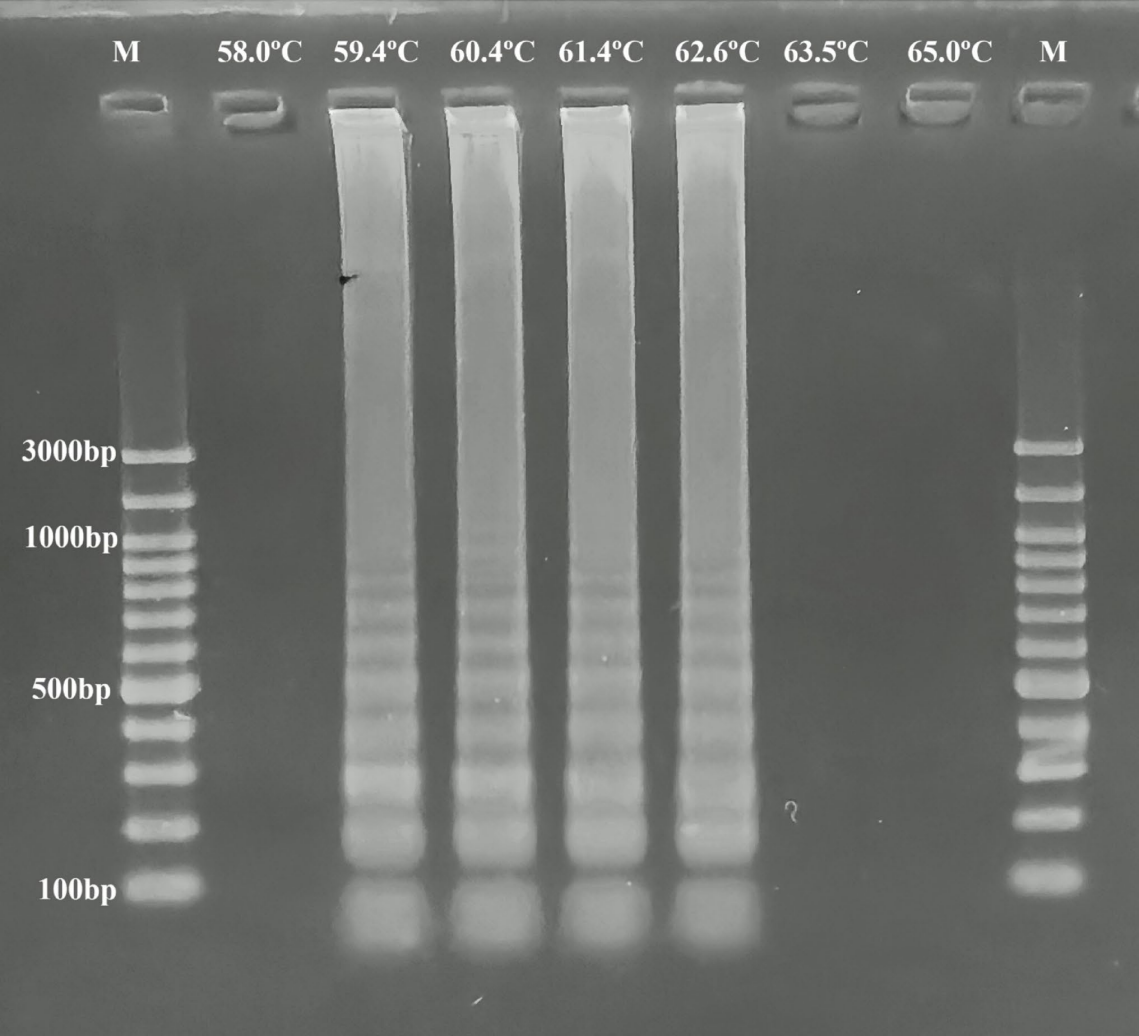
The determination of annealing temperatures of the LAMP method with agarose gel electrophoresis

In this study, the LAMP method was run for 60 min at different temperatures, and color changes (Fig. 2) and ladder-like band profiles (Fig. 3) were observed in the LAMP mixture at 59.4 °C, 60.4 °C, 61.4 °C, and 62.6 °C, and it was determined that the LAMP assay may be performed at these degrees.

**Fig. 4:**
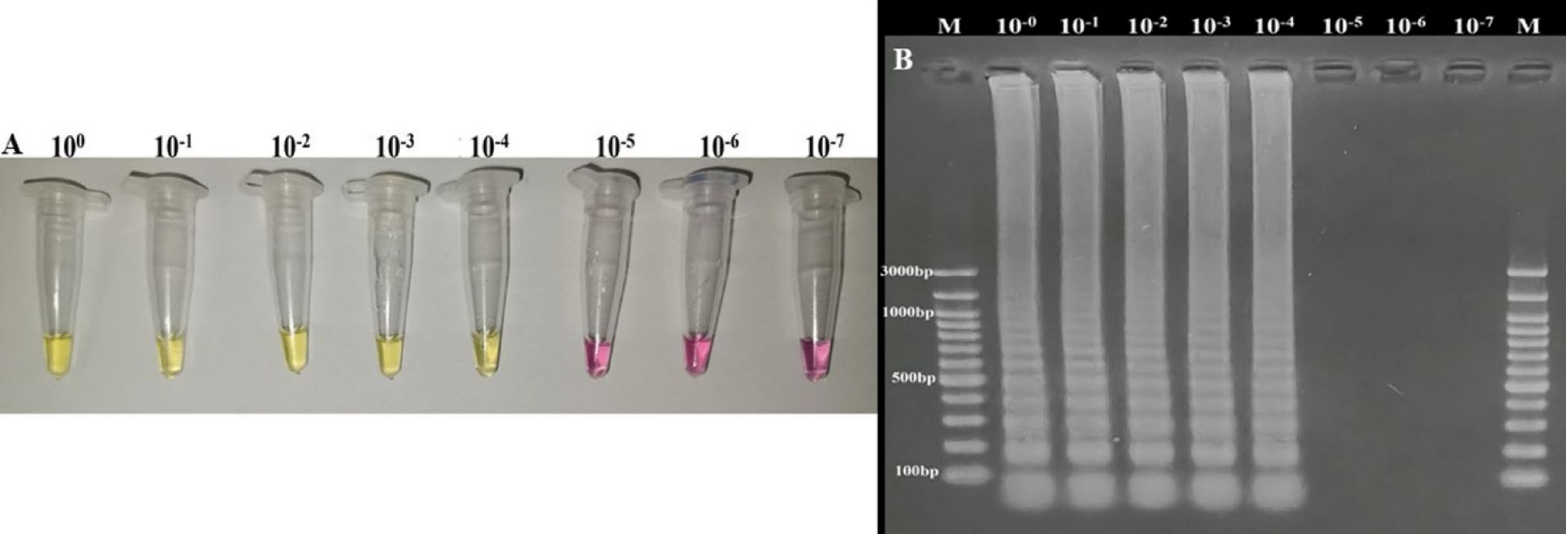
Limit of detection of LAMP assay. Color change (**A**) and ladder-like band profile (**B**) were seen in samples between 10^0^ and 10^–4^, and these samples were determined as positive, others negative

**Fig. 5:**
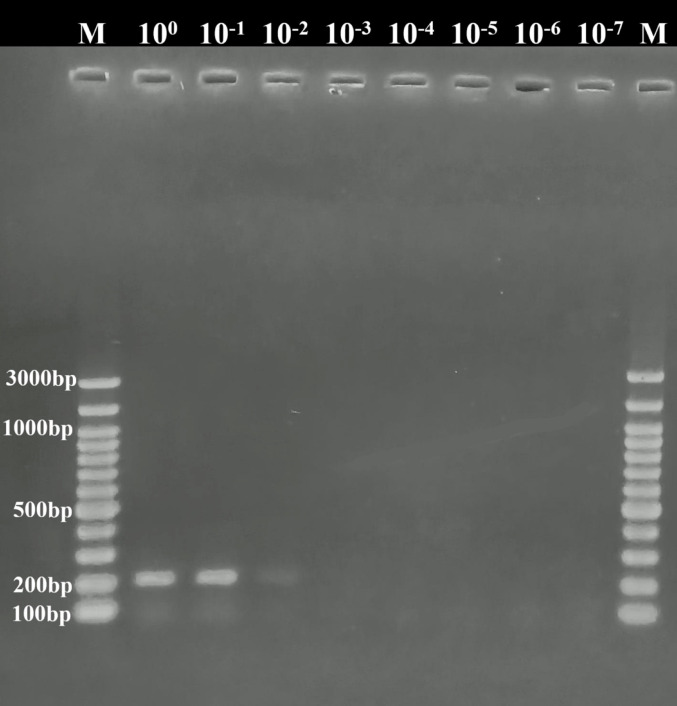
Limit of detection of *COI* gene-specific PCR assay. Samples 10^0^→10^–2^ positive, other samples negative

The limit of detection of both LAMP and PCR methods was determined, and the results were compared. For this purpose, a sample containing 47.88 ng/μL *D. immitis* DNA obtained from adult *D. immitis* was used. This sample was diluted with sterile water, and samples with different amounts of DNA (10^0^→10^–7^) were obtained. LAMP assay gave positive results in the samples up to 10^–4^ dilutions (Fig. 4), whereas PCR showed positive results in samples up to 10^–2^ dilutions (Fig. 5). These results showed that 0.0048 ng/μL of *D. immitis* DNA in the sample could be detected by LAMP, whereas 0.48 ng/μL of DNA could be identified by conventional PCR. This result indicates that the LAMP method was 100 times more sensitive than the conventional PCR method.

This study also investigated the repeatability of the LAMP assay. For this purpose, the LAMP assay was performed on three different days using samples known to be positive (n:10) and negative (n:10) samples for *D. immitis.* On all three test days, positive samples gave positive results, while negative samples were negative (supplementary figure). These results indicate that LAMP can be used for the diagnosis of *D. immitis* in hosts.

### Molecular Investigation of Blood Samples

The 300 dog and 300 cat blood samples collected from Ankara, İstanbul, and İzmir provinces were researched for *D. immitis* using both LAMP and PCR methods.

The 3% of dog blood samples (9/300) were found to be positive with the LAMP method (Table 2). *Dirofilaria immitis* was detected in all provinces with the following prevalence: 2% (2/100) of Ankara, 5% (5/100) of Istanbul, and 2% (2/100) of Izmir samples. In cat samples, *D. immitis* was identified in 0.33% (1/300). This positive sample was collected from İstanbul, but *D. immitis* was not detected in Ankara and İzmir. The difference in the prevalence of *D. immitis* in dogs (3%) and cats (0.33%) was statistically significant (*p* < 0.05).

**Table 2:** Detailed data on positive samples identified in this work

Provinces	Animal species	Sample code	Results		
			*18S rRNA-*PCR	*COI* –PCR	LAMP
Ankara	Dog	AK-KP5	+	+	+
	Dog	AK-KP14	+	+	+
İstanbul	Dog	I-KP6	*–*	*–*	+
	Dog	I-KP22	+	+	+
	Dog	I-KP59	+	+	+
	Dog	I-KP72	*–*	*–*	+
	Dog	I-KP76	+	+	+
	Cat	I-KD38	*–*	*–*	+
İzmir	Dog	IZ-KP23	*–*	*–*	+
	Dog	IZ-KP47	+	+	+

All samples were investigated with a PCR assay targeting the *18S rRNA* gene of the pathogen. *Dirofilaria immitis* was detected by PCR in 2% of samples (6/300) (Table 2). The positive samples were collected from İstanbul (3/100), Ankara (2/100), and İzmir (1/100). The pathogen was not identified in cat blood samples using PCR.

Ten samples determined to be positive by LAMP were examined using primers specific to the *COI* gene region of *D. immitis*. As a result of this examination, six samples were determined to be positive for *D. immitis* (Table 2). It was observed that the six samples determined to be positive by PCR specific to the *COI* gene region were also positive by PCR specific to the *18S rRNA* gene region (Table 2). The detailed information about positive samples were listed Table 2.

Cohen’s Kappa test was used to compare test agreement between LAMP and PCR assay results. Following the calculation process, the value was calculated as κ = 0.76. This result indicates that the level of agreement between LAMP and PCR assay results is “Substantial” (0.61–0.80).

### DNA Sequence Analysis and Phylogeny of the 18S rRNA gene of Dirofilaria immitis

In this study, the partial nucleotide sequence of the *D. immitis 18S rRNA* gene was carried out. The consensus sequences were specified and uploaded to GenBank under accession numbers: PQ496477-PQ496482. These consensus sequences had 100% nucleotide identities with each other. The 97.12–100% nucleotide similarities were seen between our *D. immitis* isolates and *D. immitis* isolates identified in various parts of the world. Furthermore, 100% nucleotide identities were present between our *D. immitis* isolates and *D. immitis* isolates reported in dogs from Türkiye (KJ183078), South Korea (FJ799911, FJ799916, and FJ799917), and Argentina (HM124350), and in mosquito species in Argentina (HM124347-HM124349).

**Fig. 6:**
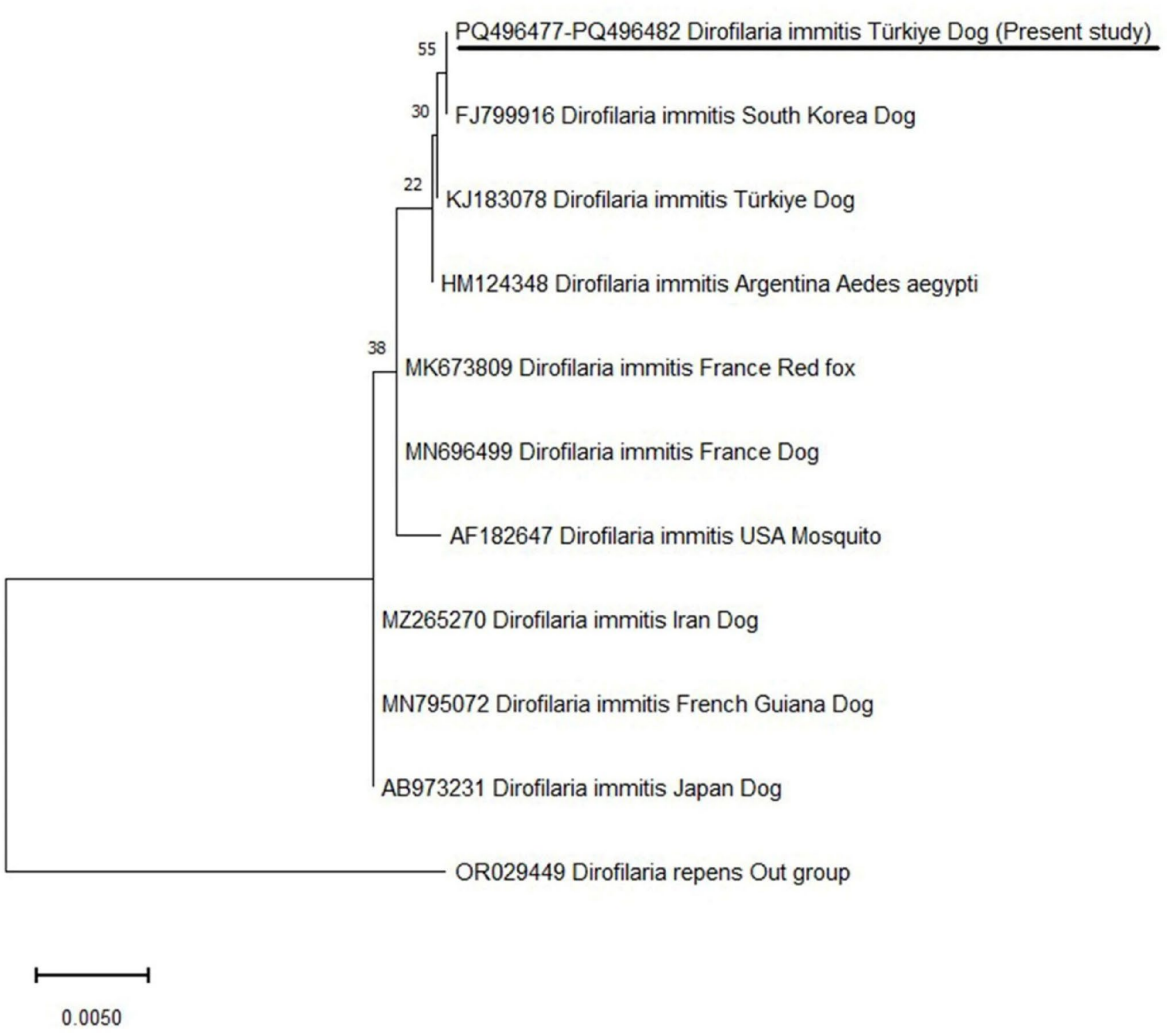
Phylogenetic tree based on the *18S rRNA* gene sequence of *D. immitis* (GenBank accession numbers: PQ496477-PQ496482) using the maximum likelihood method. The phylogenetic tree was inferred using the Tamura-3 paramater model (27). The scale bar represents 0.01 substitutions per nucleotide position. *D. repens* (JB837173) was used as an outgroup. Evolutionary analyses were performed in MEGA-11 (21)

In the phylogenetic tree, our *D. immitis* isolates were placed with same branch the *D. immitis* isolates identified in different parts of the world (Fig. 6).

The phylogenetic analyses of the *18S rRNA* gene of *D. immitis* isolates revealed that single-nucleotide polymorphisms (SNPs) were present between these isolates. While the 584th nucleotide of *D. immitis* identified in the study and *D. immitis* detected in the USA (AF182647) and South Korea (FJ799916) was adenine, the same nucleotide was guanine in France (MN696499 and MK673809), French Guiana (MN795072), Japan (AB973231), and Iran (MZ265270) *D. immitis* isolates. The 996th nucleotide in our *D. immitis* isolates and *D. immitis* reported in the USA (AF182647), South Korea (FJ799916), Türkiye (KJ183078), and France (MN696499 and MK673809) was cytosine, whereas this nucleotide in *D. immitis* isolates from French Guiana (MN795072), Japan (AB973231), and Iran (MZ265270) was thymine.

## Discussion

*Dirofilaria immitis* is an important nematode species identified in various hosts in almost all regions of the world and transmitted to hosts by vector mosquitoes [6, 7]. Recent studies have indicated an increase in the areas at risk regarding *D. immitis*. This increase may be linked to several factors, including an increase in seasonal temperatures due to climate change, the expansion of the mosquito's habitat and the increase in its abundance in these areas, and the trade of dogs between countries (especially from endemic areas) [2, 6, 7, 30]. Conventional, serological, and molecular methods have been used for the identification of *D. immitis* in hosts [9]. Molecular-based tools have been preferred by researchers due to their high sensitivity and specificity [6, 31–33]. However, studies have shown that results can differ even when the same samples are analyzed by different molecular methods. Researchers have claimed that this difference may be related to the amount of pathogen DNA in the sample [34, 35]. For this reason, various methods have been developed with higher specificity and sensitivity, such as LAMP [11]. In this work, the LAMP method was developed to identify *D. immitis* in hosts. The limit of detection of the LAMP method were determined, and the results were compared with the PCR detection limit. The obtained data revealed that the LAMP assay was about 100 times more sensitive than the PCR method (Figs. 4, 5). We believe that the LAMP method applied in our study will allow more accurate results to be obtained in epidemiological studies and will contribute to the health of cats and dogs by being applicable in pet clinics.

One of the most important definitive hosts of *D. immitis* is domestic dogs, therefore, lots of studies have been conducted to determine *D. immitis* the prevalence in dogs using molecular techniques [6]. In these studies, *D. immitis* was identified with different prevalence, such as 1.75% in Spain [36], 2.18% in Slovakia [37], 6.15% in Romania [38], 0.2% in Germany [32], 25.4% in Serbia [39], 0.86% in Austria [40], 22.5% in Costa Rica [41], 8% in Mexico [42], 14.5% in Tunisia [43], 0.9% in Algeria [44], 24% in China [31], 7.08% in Taiwan [45], 4.45% in Iran [46], 0.29% in Kyrgyzstan [47], and 5.6% in Hong Kong [48]. Türkiye has a suitable climate and geographical features for vector species, such as mosquitoes, to survive and reproduce [14–18]. It is known that *D. immitis* is an endemic species in the country [14, 19, 49–51], and the first record of the presence of this nematode in Türkiye dates back to 1951 [52]. After the first report, *D. immitis* was researched using several methods, such as necropsy, microscopic, serological, and molecular methods, and in these studies, the prevalence of *D. immitis* varied between 0 and 50.8% among dogs obtained from various provinces [19, 20, 53–58]. In this study, *D. immitis* was identified in 3% (9/300) of the samples with LAMP, whereas the prevalence was 2% (6/300) with PCR. As *D. immitis* is a mosquito-borne helminth, its prevalence may vary according to the climatic conditions of the sampling areas, seasonal temperature levels, rainfall patterns, economic status of the country, and the date of sample collection in the studies. Furthermore, the prevalence of *D. immitis* may vary according to the number of animals included in the study, their age, sex, housing status (indoor or outdoor), whether they are owned or stray, and the specificity and sensitivity of the diagnostic methods preferred in the studies [6, 7, 19, 35, 44, 55, 58–60]. All dogs included in this study are owned dogs and are routinely monitored by veterinarians. It is considered that the differences in the prevalence of *D. immitis* reported in studies from various countries, including this study, may be due to one or more of the above-mentioned reasons.

*Dirofilaria immitis* can cause mild to severe infection in cats [8]. This nematode has been reported in South Korea [61, 62], Italy [63], Iran [64], Thailand [65, 66], China [67], USA [33, 35] using molecular methods among cats. In Türkiye, there is a lack of data on the prevalence of *D. immitis*. In the country, only three studies have been carried out to investigate *D. immitis* in cats, and the pathogen could not be detected by microscopic and molecular methods [56, 68, 69]. However, seropositivity was reported in cats in studies conducted in the Aegean region [69] and Kars province [70]. In this work, 300 cat blood samples obtained from İstanbul, Ankara, and İzmir were researched for *D. immitis* using LAMP and PCR methods. *Dirofilaria immitis* DNA was detected in one cat collected from İstanbul by the LAMP method, but no positivity was seen with both *18S rRNA* and *COI* genes-specific PCR (Table 2). This is the first molecular report of *D. immitis* in cats in Türkiye. Studies performed in the world and Türkiye show that the prevalence of *D. immitis* may vary depending on the host species. For instance, studies conducted in the USA [33], Hong Kong [48], and Türkiye [56] reported that the prevalence of *D. immitis* was higher in dogs than in cats. Similar results were found in this study, and the prevalence of *D. immitis* was 3% in dogs and 0.33% in cats. There may be more than one reason for the difference in the prevalence of *D. immitis* in dogs and cats, but the most important ones are; (i) cats are smaller in size compared to dogs and are less exposed to mosquitoes compared to dogs, (ii) the life span of microfilariae and adult forms of *D. immitis* is approximately two times shorter in cats compared to dogs, and (iii) cats are more resistant to *D. immitis* compared to dogs [2, 7, 8, 33, 35].

Within the aim of the study, 600 samples were examined for *D. immitis* using both LAMP and PCR assays. The LAMP and PCR results revealed that four samples (three dog and one cat sample) that were positive by LAMP were negative by PCR. All samples that were positive by PCR were also positive by LAMP (Table 2). Cohen's kappa was used to calculate the level of agreement between LAMP and PCR tests, the value was κ = 0.76. Moreover, it has also been observed that the value is close to the upper limit of the “Substantial” level (0.61–0.80) [29]. This indicates that the agreement between the tests is quite strong. These results, along with other advantages of the LAMP test (such as not requiring expensive equipment to perform the test and the ability to obtain rapid results), demonstrate that it can be used as a diagnostic test for *D. immitis* in hosts in the field.

In the studies, DNA sequence analysis is preferred for several reasons, such as; to confirm the results of molecular diagnostic methods, to perform phylogenetic analyses of pathogens and to reveal their genetic diversity, to understand their life cycles, to identify new host species, and to reveal new geographical regions at risk for pathogens in the studies [15, 16, 71–73]. Several studies have been conducted for molecular characterization and phylogenetic analyses of *D. immitis* targeting different gene regions, such as *18S rRNA, COI, NADH, 12S rRNA, 5.8S, ITS-2,* and *28S rRNA* [17–19, 73]. In this work, the *18S rRNA* gene region of all PCR-positive samples was sequenced, and 100% nucleotide identities were present between the obtained consensus sequences. BLASTn analyses determined that 97.12–100% was present between our *D. immitis* isolates and *D. immitis* identified in different parts of the world. Phylogenetic analysis of the *18S rRNA* gene region of *D. immitis* isolates indicated the presence of SNPs in the 584th (A→G) and 996th (C→T) nucleotides of the gene. Studies have shown that the *18S rRNA* gene is conserved, and mutations in this gene region are very rare. Therefore, it has been reported that mutations in the *18S rRNA* gene region of the same species of pathogens may result in genotypic differences [3, 74]. In this study, SNPs were detected among *D. immitis* isolates, including ours, therefore, it is considered that different *D. immitis* genotypes circulate in hosts. In addition, the molecular and phylogenetic data obtained in this study will contribute to the studies to determine the genotypes of *D. immitis* in Türkiye and the world.

The limitation of this study is that the dog and cat blood samples researched molecularly within the aim of the study could not be examined microscopically for *D. immitis*. Therefore, the sensitivity of LAMP and PCR assays could not be correlated with the number of microfilariae per mL of blood.

## Conclusion

The LAMP method for identifying *D. immitis* among dogs and cats was developed and optimized in the current work. Furthermore, the prevalence of *D. immitis* was researched using molecular methods (LAMP and PCR), and the nematode was identified in both dogs and cats. *Dirofilaria immitis* was identified in 2% of dogs using PCR, while no positivity was seen in cat samples. However, *D. immitis* was detected in 3% of dogs and 0.33% of cat samples with the LAMP assay. These results show that LAMP is more sensitive than PCR for diagnosing *D. immitis*. It is known that *D. immitis* is a zoonotic species of nematode that is transmitted by mosquitoes and that this nematode can cause mild to severe infections in animals and humans. *Dirofilaria immitis* was detected in all provinces where samples were collected as part of the study. Considering that these provinces have the largest populations of both pets (dog and cat) and humans in Türkiye, it is assessed that people and animals in these regions are at risk from the pathogen. This study has molecularly demonstrated the presence of *D. immitis* in three major provinces of Türkiye. However, it is considered that samples should be collected from more provinces and investigated for pathogens using methods with higher sensitivity and specificity, such as LAMP, to obtain more comprehensive molecular results at the national level.

## Supplementary Information

Below is the link to the electronic supplementary material.

Supplementary Material 1.

Supplementary Material 2.

## Data Availability

All data generated or analyzed during this study are included in this manuscript.
