# Supplementary material for: Application of COI-LAMP for Detection of Dirofilaria immitis with High Sensitivity and Specificity in Epidemiological Studies
Source: Acta Parasitol. 2026 Mar 9;71(2):58. doi: 10.1007/s11686-026-01253-w (PMC12971837; doi:10.1007/s11686-026-01253-w)
Supplement: Supplementary file 1 — Supplementary Material 1. [file 11686_2026_1253_MOESM1_ESM.docx]

**Supplementary Table. Information on *D. immitis* isolates used in the design of LAMP primers**

| **GenBank Accession number** | **Country** | **Host** |
| --- | --- | --- |
| OQ726801 | Greece | Dog |
| PV469776 | Spain | Dog |
| AM749229 | Italy | Dog |
| MK250742 | Thailand | Dog |
| KF918394 | South Korea | Cat |
| KF918395 | South Korea | Cat |
| KF918398 | South Korea | Cat |
| MW577348 | Thailand | Human |
| HQ540424 | Brazil | Human |
| EU169124 | China | Red panda |
