# Supplementary material for: Application of COI-LAMP for Detection of Dirofilaria immitis with High Sensitivity and Specificity in Epidemiological Studies
Source: Acta Parasitol. 2026 Mar 9;71(2):58. doi: 10.1007/s11686-026-01253-w (PMC12971837; doi:10.1007/s11686-026-01253-w)
Supplement: Supplementary file 2 — Supplementary Material 2. [file 11686_2026_1253_MOESM2_ESM.docx]

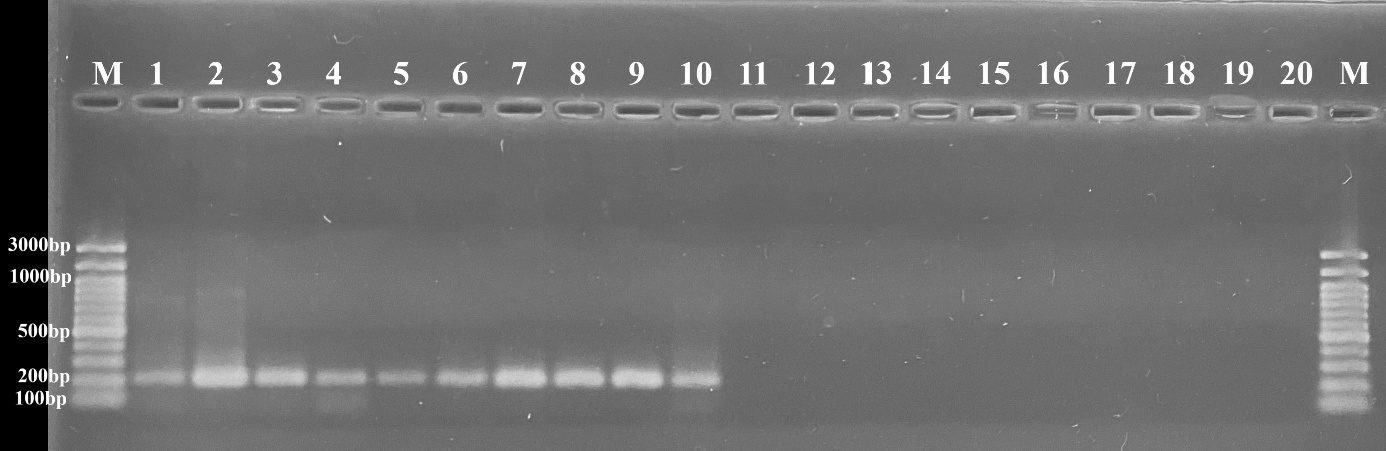


**Supplementary Fig.1. COI PCR assay of *D. immitis* positive and negative samples. M. Marker, 1-10. *D. immitis* positive samples, 11-20. *D. immitis* negative samples.**

**
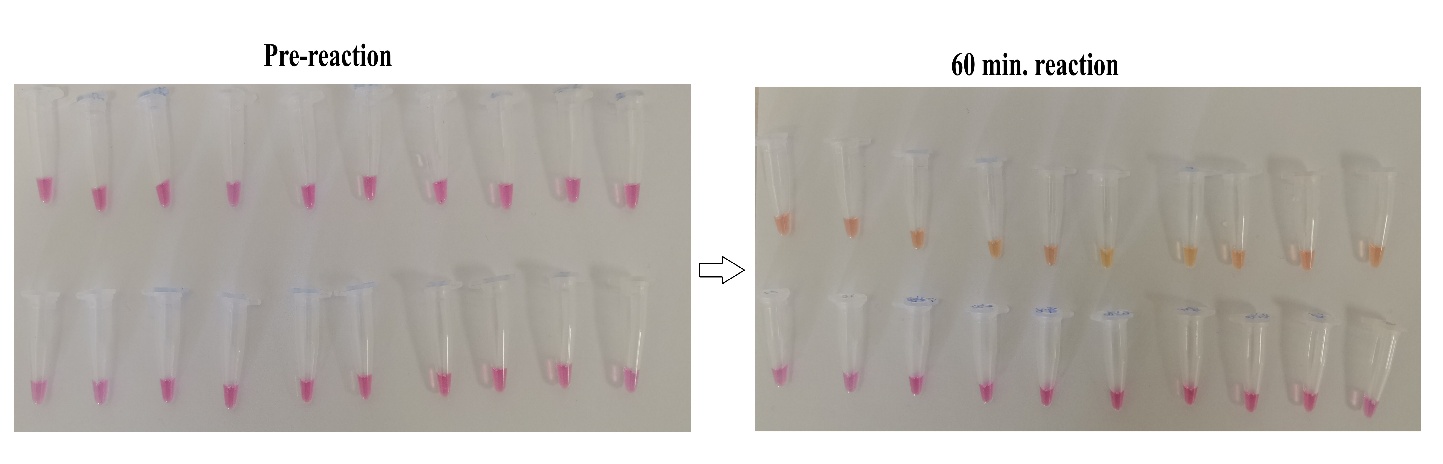
**

**Supplementary Fig. 2. Day-1 LAMP assay results of *D. immitis* positive and negative samples. After LAMP reaction color change was seen in positive samples.**

**
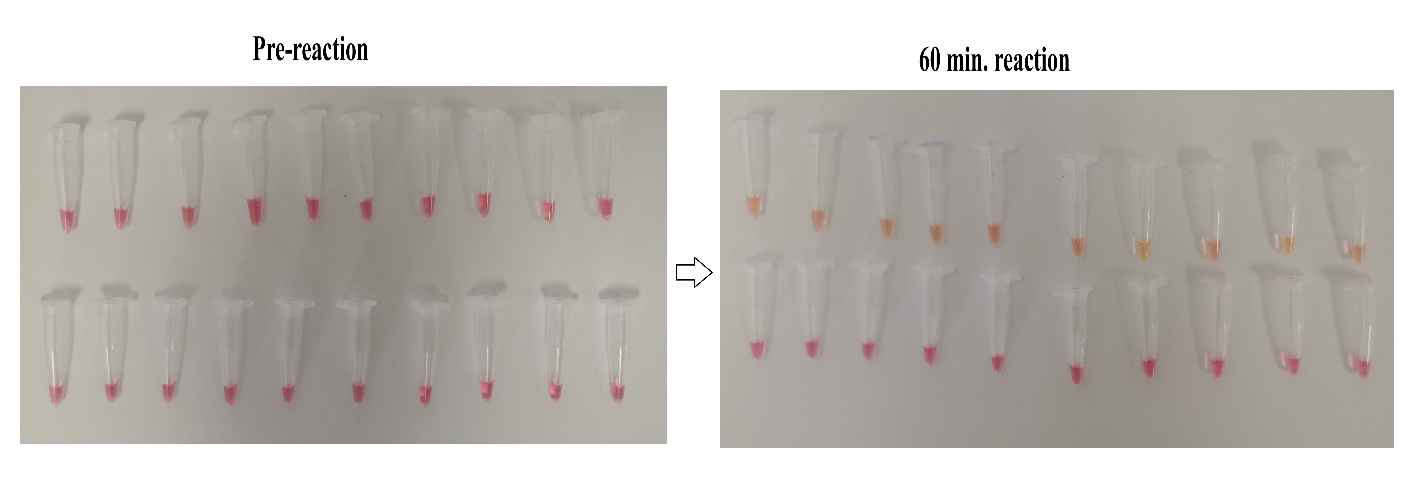
**

**Supplementary Fig. 3. Day-2 LAMP assay results of *D. immitis* positive and negative samples. After LAMP reaction color change was seen in positive samples.**

**
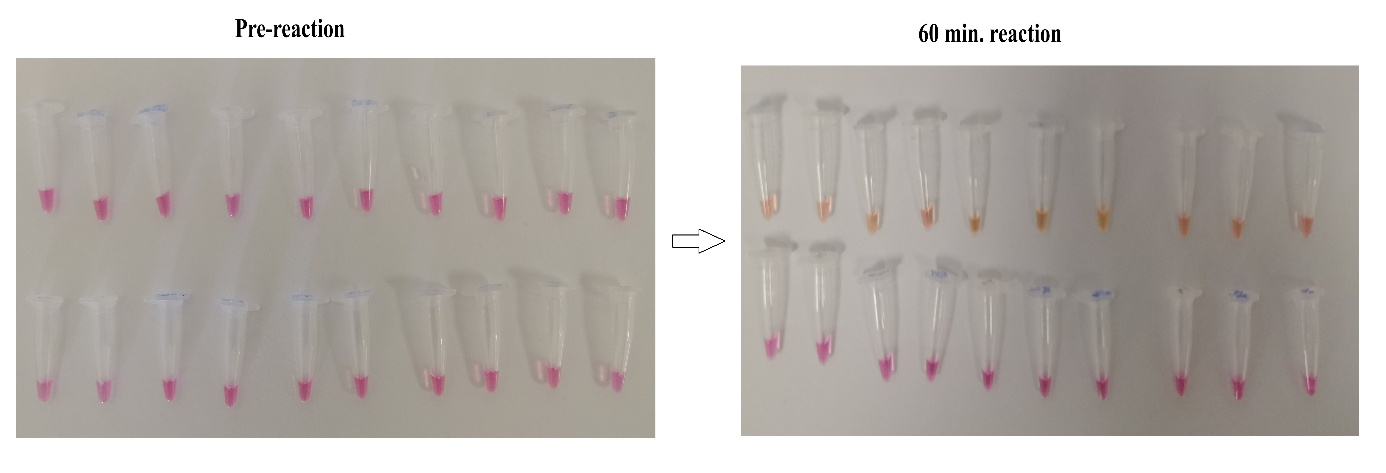
**

**Supplementary Fig. 4. Day-4 LAMP assay results of *D. immitis* positive and negative samples. After LAMP reaction color change was seen in positive samples.**
